# A new method for evaluating lung volume: AI-3D reconstruction

**DOI:** 10.3389/fphys.2023.1217411

**Published:** 2023-09-14

**Authors:** Wang Rui, Shang Yuhang, Li Yang, Yang Yue, Tang Ze, Zhao Yujie, Ma Xiaochao, Qin Da, Cui Youbin, Lu Tianyu

**Affiliations:** ^1^ Department of Thoracic Surgery, The First Hospital of Jilin University, Changchun, China; ^2^ School of Public Health, Jilin University, Changchun, China; ^3^ Department of Critical Medicine, The First Affiliated Hospital of Jiamusi University, Jiamusi, China

**Keywords:** lung volume calculation, AI-3D reconstruction, ICC, donor-recipient lung volume matching, lung transplantation

## Abstract

**Objective:** This study aims to explore the clinical application of an AI-3D reconstruction system in measuring lung volume and analyze its practical value in donor-recipient size matching in lung transplantation.

**Methods:** The study retrospectively collected data from 75 subjects who underwent a plethysmography examination and lung CT at the First Hospital of Jilin University. General data and information related to lung function, and imaging results were collected. The correlation between actual total lung volume (aTLV), predicted total lung volume (pTLV), and artificial intelligence three-dimensional reconstruction CT lung volume (AI-3DCTVol) was analyzed for the overall, male, and female groups. The correlation coefficient and the absolute error percentage with pTLV and AI-3DCTVol were obtained.

**Results:** In the overall, male, and female groups, there were statistical differences (*p* <0.05) between the pTLV formula and AI-3D reconstruction compared to the plethysmography examination value. The ICC between pTLV and aTLV for all study participants was 0.788 (95% CI: 0.515–0.893), *p* <0.001. Additionally, the ICC value between AI-3D reconstruction and aTLV was 0.792 (95% CI: 0.681–0.866), *p* <0.001. For male study participants, the ICC between pTLV and aTLV was 0.330 (95% CI: 0.032–0.617), *p* = 0.006. Similarly, the ICC value between AI-3D reconstruction and aTLV was 0.413 (95% CI: 0.089–0.662), *p* = 0.007. In the case of female research subjects, the ICC between pTLV and aTLV was 0.279 (95% CI: 0.001–0.523), *p* = 0.012. Further, the ICC value between AI-3D reconstruction and aTLV was 0.615 (95% CI: 0.561–0.870), *p* <0.001.

**Conclusion:** The AI-3D reconstruction, as a convenient method, has significant potential for application in lung transplantation.

## Introduction

The evaluation of lung volume has numerous clinical applications, including the assessment of lung function and treatment of chronic diseases, which are crucial for managing patient care effectively (Kojima et al., 2011). The lung volume calculation can help assess patients’ lung function, diagnose diseases and monitor the progression, evaluate surgical risks, and provide auxiliary references for the formulation of diagnosis and treatment plans (Milner, 2000). In recent years, surgical techniques of lung transplantation have made significant progress due to technical advancements in organ transplantation (Valapour et al., 2015; Weill, 2016; Chambers et al., 2018). Accurate lung volume assessment has been increasingly important in lung transplantations, leading to improved organ-matching efficiency and better recipient outcomes (Eberlein et al., 2012a; Eberlein et al., 2012b; Eberlein et al., 2015). Several primary methods such as lung function testing, radiological examination, electrical impedance tomography, and plethysmography are currently available to determine lung volumes (Wolf and Arnold, 2005). Developed by Dubois et al., in 1956, the plethysmography examination measures the residual volume (RV) and the total lung volume (TLV) in the chest cavity based on Boyle’s law (Dubois et al., 1956). The plethysmography examination of lung function testing is considered the most accurate method for lung volume assessment (Goldman and Becklake, 1959). Currently, the lung function evaluation for donor patients with brain death heavily relies on the predicted total lung volume (pTLV) formula, which is developed based on a small group sample from the non-Chinese population (Stocks and Quanjer, 1995; Clayton, 2007). Considering the vast population size of China, the need of a lung volume measurement explicitly designed for the Chinese population is highly demanded (Elmaleh-Sachs et al., 2022; McCormack et al., 2022).

For the past few years, artificial intelligence three-dimensional reconstruction (AI-3D) technology has been developed and applied to evaluate lung and digestive system diseases (Chan et al., 2015; Rahaman et al., 2020; Barmpoutis et al., 2022). The AI-3D reconstruction utilizes CT, MRI, and other scanning technologies to obtain continuous body structure data. Relevant software is used to recognize and process the image data prior to the construction of a virtual three-dimensional model of human organs. Compared to two-dimensional images, three-dimensional models are more intuitive, easier to observe, and provide better visualization of the characteristics and abnormalities of relevant tissues (Wang et al., 2011). Expanding from the outstanding potential of AI-3D reconstruction in our team’s previous research, this study utilizes AI-3D reconstruction technology to process CT image data of the subjects, and acquire lung volume data to ultimately conduct a comparative analysis with traditional calculation formulas and plethysmography examination (Cai et al., 2018; Ma et al., 2023).

## Materials and method

Based on the inclusion and exclusion criteria, this study retrospectively collected data from 76 patients who had undergone plethysmography examination and lung CT at the Thoracic Surgery Department of the First Hospital of Jilin University from March 2021 to December 2022. Prior to the examination, every patient undergoes respiratory training to ensure their ability to cooperate with the plethysmography examination. The study also collected the patient’s sex, age, height, weight, and medical history. The Ethics Committee of the First Hospital of Jilin University approved this study (2021-069).

### Inclusion criteria

(1) Subjects aged between 18 and 70 years old.
(2) Subjects who have not undergone chest surgery and who do not have severe spinal or thoracic deformities.
(3) Subjects who have normal heart function indicated by echocardiography.
(4) Subjects who can complete a plethysmography examination after respiratory training.

### Exclusion criteria

(1) Subjects with a history of chronic lung disease that causes diffuse symptoms in the chest, such as extensive pneumonia and pleural effusion.
(2) Subjects with a history of lung, mediastinal mass, or heart disease or smoking history.
(3) Subjects whose lung CT examination was not performed within 72 h of the physical examination.
(4) Subjects with obstructive ventilation dysfunction, as indicated by an FEV1/FVC (%) of less than 70.
(5) Subjects with restrictive ventilation dysfunction, indicated by a measured/predicted VC or TLV (%) of less than 80.

## Plethysmography examination

The examination process followed the plethysmography lung volume and airway resistance examination guidelines set by the Professional Group for Pulmonary Function of the Respiratory Branch of the Chinese Medical Association in 2015 (Association and CMARD, 2015). A master Screen Body plethysmograph (JAEGER Master Screen Body) was used. The testing was conducted 0.5–2 h after eating, and subjects were instructed not to have abdominal distension and to avoid medication such as corticosteroids and bronchodilators that could affect respiratory functions for at least 6 h before testing. The instrument was calibrated for atmospheric pressure and temperature prior to the first inspection on the same day to ensure the accuracy of the inspection. Subjects were also instructed not to wear tight clothing or belts that could restrict chest movement, and prior to testing, they were asked to clean their nasal and oral secretions. Subsequently, the technician explained the testing procedures and precautions, then the subjects entered the physical examination box and adjusted the seat height to a level that the subjects were able to use the mouthpiece without bending or extending their neck. After that, the box was closed, and the physical examination was conducted under the technician’s guidance. Data was collected for three to five sets of tests, with the difference between each set ranging from 0% to 10%, and groups with a coefficient of variation greater than 10% were eliminated manually.

## Lung volume calculation formula

This study utilizes the prediction equations of the European Community for Steel and Coal (ECSC), (Quanjer, 1983), presented as follows:

Male:pTLV=7.99 Height−7.08

Female:pTLV=6.60 Height−5.79

### Evaluation of lung volume using AI-3D reconstruction

**FIGURE 1:**
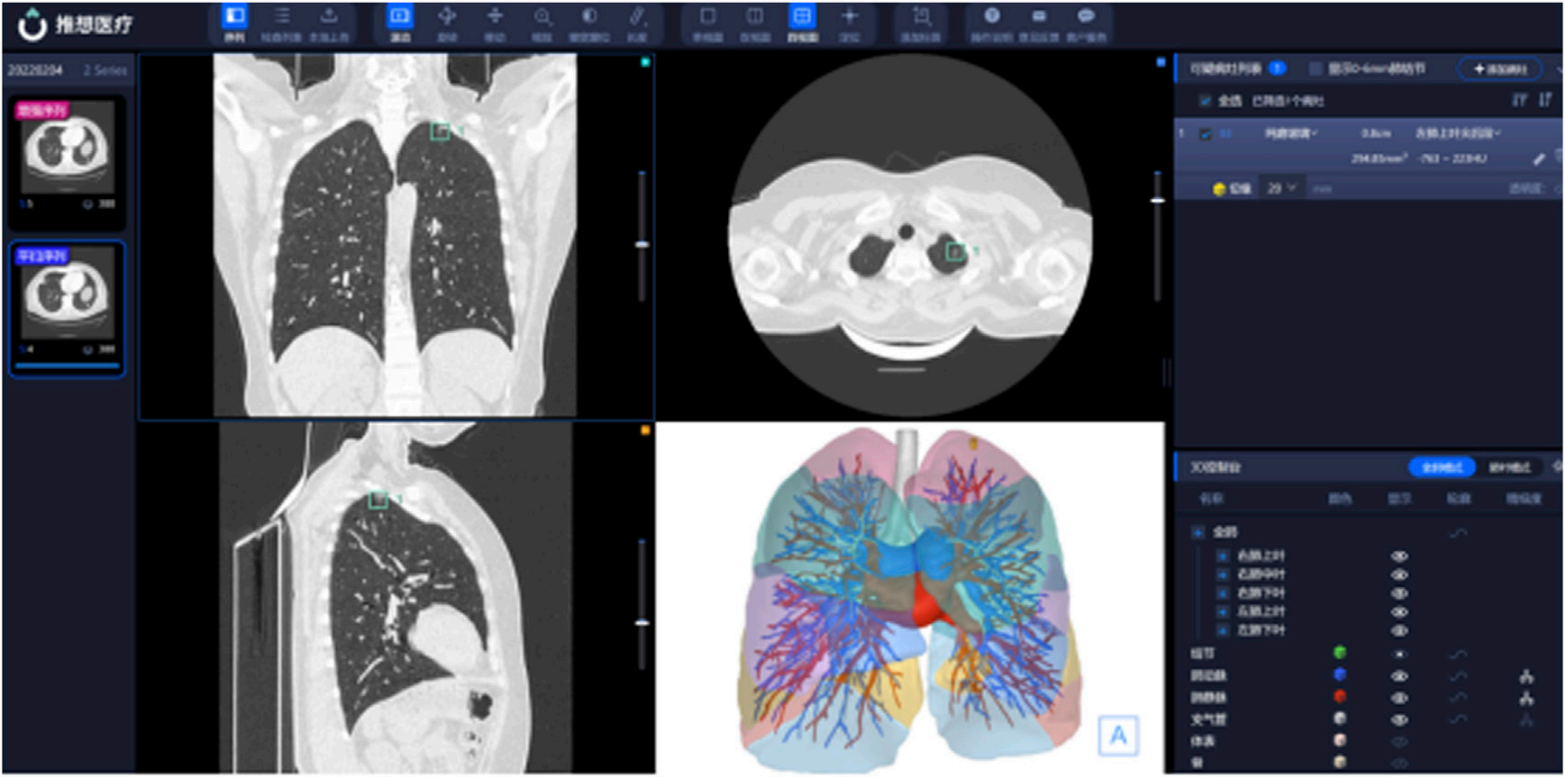
Human-computer interaction interface diagram.

To achieve optimal reconstruction results, the CT imaging data used in the evaluation process are carefully selected with specific scanning parameters, including a layer thickness of 1 mm, a minimum of 100 images, image continuity, and the inclusion of the complete lung area in the scanning area (512 × 512 pixels minimum) with no observation of breathing artifacts or foreign objects. The lung window and mediastinal data that meet the aforementioned conditions are compressed in the zip format and uploaded to the system of Infervisual AI-3D reconstruction V1, which autonomously desensitizes patient information and segments and reconstructs the lung lobes, trachea, pulmonary arteries, veins, and nodules (Figure 1). The entire model reconstruction is completed by AI independently, without any manual intervention (Xu et al., 2023).

### Error analysis

Plethysmography examination is conducted with the person in an upright position, whereas a CT examination is conducted with the person in a supine position. Due to the effect of gravity, the position of the diaphragm can change between the upright and supine positions could lead to variations in lung volume compliance. To control an average increase of 10% in actual total lung volume (aTLV) in the standing position compared to the supine position, we utilize the subsequent two equations for calibration (Yamada et al., 2020).

$$\left|\left(\text{pTLV}-\text{aTLV}\right)/\text{aTLV}\right|\times 100\%$$

$$\left|\left(\text{AI}-3\text{DCTVol}-\text{aTLV}\right)/\text{aTLV}\right|\times 100\%$$

## Statistical analysis

The data analysis was conducted using SPSS 26.0 and R software (4.2.2). Rates and component ratios were utilized to present counting data. To test the balance of general data, one-way ANOVA was applied for quantitative data. Mean ± standard deviation was used to express continuous variables that conform to a normal distribution. The independent sample *t*-test was used to evaluate continuous variables in two groups that conform to a normal distribution. Comparisons between multiple groups were conducted through an analysis of variance. In the case of continuous variables conforming to normal distribution within a group, a paired *t*-test was employed. Data that do not exhibit a normal distribution are organized using the median-quartile interval (P25-P75), and statistical analysis is conducted through non-parametric tests. The intra-group correlation coefficient is used to analyze the correlation between different measurement methods. A *p*-value less than 0.05 indicates statistical significance.

## Result

**FIGURE 2:**
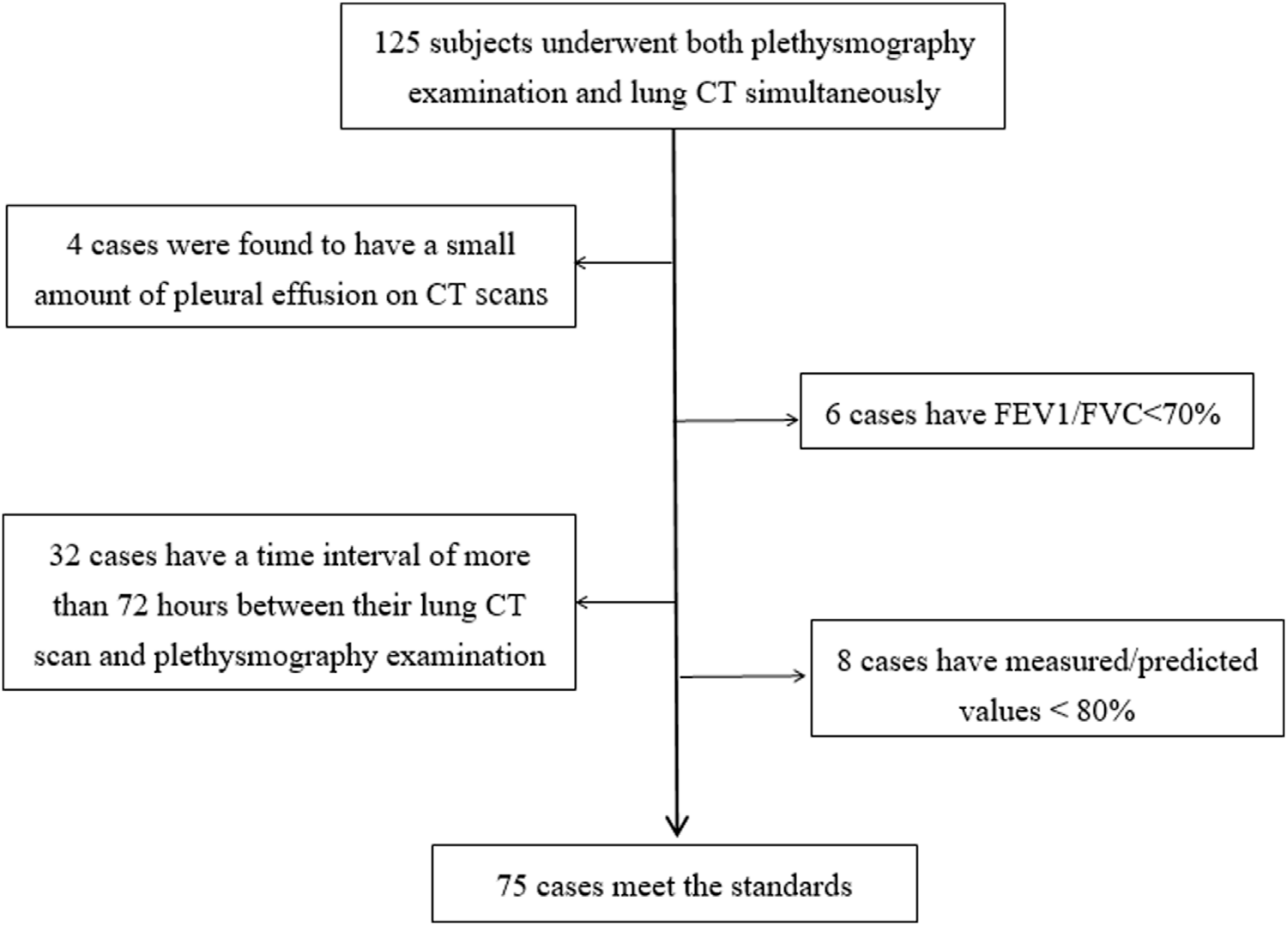
Research object inclusion process.

According to the inclusion and exclusion criteria, we collected a sample of 125 subjects who underwent a plethysmography examination and lung CT scan at the Thoracic Surgery Department of the First Hospital of Jilin University between March 2021 and December 2022. After the selection process indicated in Figure 2, we included 75 eligible subjects, consisting of 31 males and 44 females. The basic information about the patients is presented in Table 1.

**TABLE 1:** Basic information of research subjects.

Characters	All (*n* = 75)	Male (*n* = 31,41.33%)	Female (*n* = 44,58.67%)
Age, year	56.69 ± 9.41	59.65 ± 9.65	54.61 ± 8.76
Height, m	1.65 ± 0.07	1.72 ± 0.50	1.60 ± 0.04
Weight, kg	63.33 ± 9.76	69.27 ± 10.36	59.14 ± 6.75
BMI, kg/m^2^	23.28 ± 2.74	23.41 ± 3.10	23.19 ± 2.49
aTLV, L	5.15 ± 1.00	6.09 ± 0.76	4.49 ± 0.48
pTLV, L	5.53 ± 1.00	6.65 ± 0.40	4.75 ± 0.23
AI-3DCTVol, L	4.98 ± 0.95	5.87 ± 0.62	4.36 ± 0.55

In the overall, male, and female groups, there were statistical differences (*p* <0.05) between the pTLV formula and AI-3D reconstruction compared to the plethysmography examination. The mean pTLV in all three groups was greater than aTLV, and the mean aTLV was greater than AI-3DCTVol, as shown in Table 2 and Table 3.

**TABLE 2:** Comparison of pTLV and plethysmography examination.

Groups	aTLV	pTLV	t	*p*
Overall	5.15 ± 1.00	5.53 ± 1.00	−6.232	0.001
Male	6.09 ± 0.76	6.65 ± 0.40	−5.010	0.001
Female	4.49 ± 0.48	4.75 ± 0.23	−4.051	0.001

**TABLE 3:** Comparison of AI-3DCTVol and plethysmography examination.

Groups	aTLV	AI-3DCTVol	t	*p*
Overall	5.15 ± 1.00	4.98 ± 0.95	−2.936	0.004
Male	6.09 ± 0.76	5.87 ± 0.62	−2.082	0.046
Female	4.49 ± 0.48	4.36 ± 0.55	2.065	0.045

**FIGURE 3:**
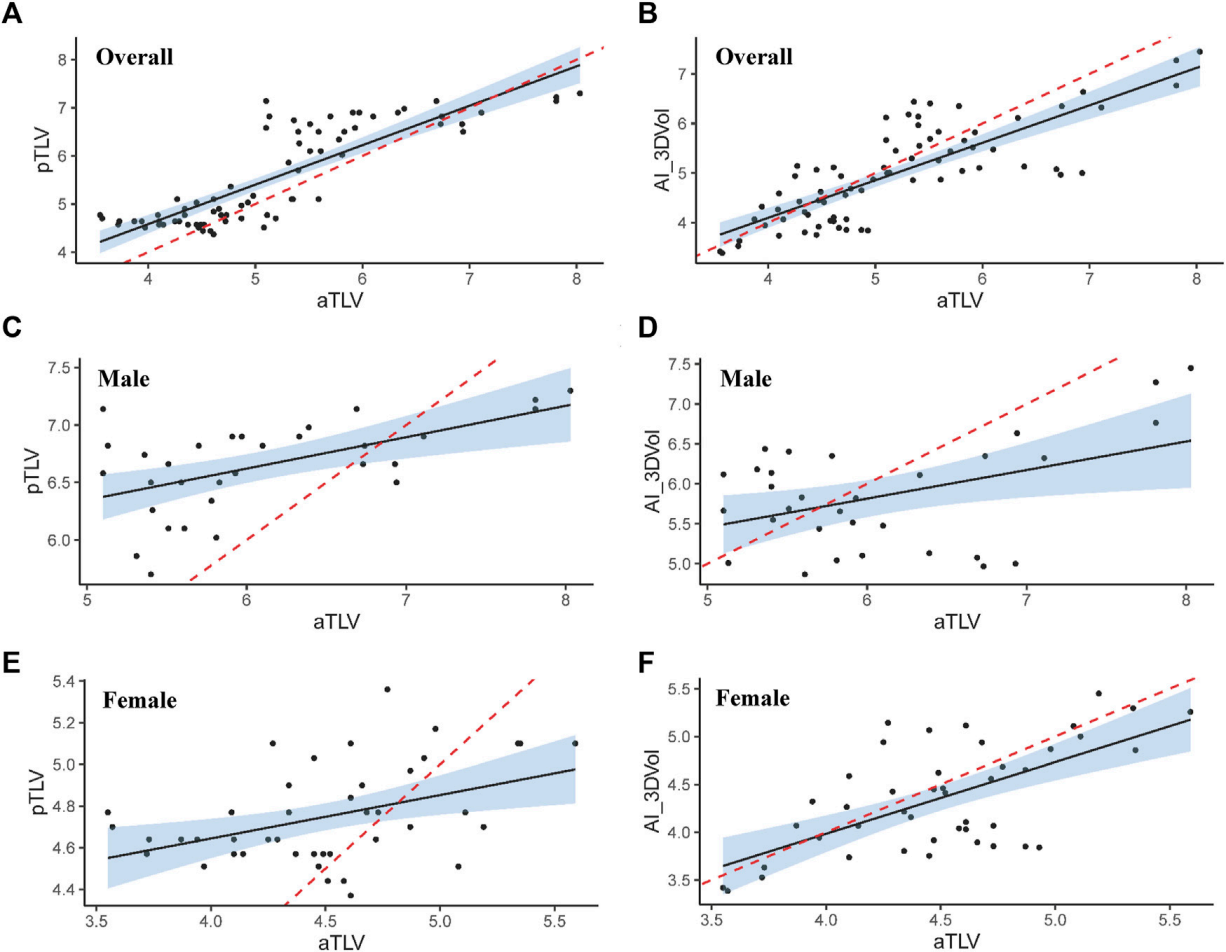
The overall correlation between aTLV and pTLV **(A)**, aTLV and AI-3DCTVol **(B)**; Correlation between male aTLV and pTLV **(C)**, aTLV and AI-3DCTVol **(D)**; Correlation between aTLV and pTLV **(E)**, aTLV and AI-3DCTVol **(F)** (the red dashed line serves as the identification line).

The intra-group correlation coefficient (ICC) between pTLV and aTLV for all study participants was 0.788 (95% CI: 0.515–0.893), *p* <0.001. Additionally, the ICC value between AI-3D reconstruction and aTLV was 0.792 (95% CI: 0.681–0.866), *p* <0.001. Notably, the value obtained by AI-3D reconstruction is more similar to the aTLV value. For male study participants, the ICC between pTLV and aTLV was 0.330 (95% CI: 0.032–0.617), *p* = 0.006, and the ICC value between AI-3D reconstruction and aTLV was 0.413 (95% CI: 0.089–0.662), *p* = 0.007, which more closely approximates the aTLV value compared to the pTLV method. In the case of female research subjects, the ICC between pTLV and aTLV was 0.279 (95% CI: 0.001–0.523), *p* = 0.012, and the ICC value between 3D reconstruction and aTLV was 0.615 (95% CI: 0.561–0.870), *p* <0.001. Similarly, the AI-3D reconstruction value aligns more with the aTLV value in this case. The specific correlation situation is illustrated in Table 4 and Figure 3.

**TABLE 4:** Correlation coefficients between aTLV and pTLV, aTLV and AI-3DCTVol.

Characters	pTLV and aTLV		AI-3DCTVol and aTLV	
	ICC(95%CI)	*p*	ICC(95%CI)	*p*
Overall	0.788 (0.515–0.893)	<0.001	0.792 (0.681–0.866)	<0.001
Male	0.330 (0.032–0.617)	0.006	0.413 (0.089–0.662)	0.007
Female	0.279 (0.001–0.523)	0.012	0.615 (0.516–0.870)	<0.001

## Absolute error percentage analysis of aTLV and pTLV, aTLV and AI-3DCTVol

The percentage of absolute error of pTLV and AI-3DCTVol is calculated as follows:

$$\left|\left(\text{pTLV}-\text{aTLV}\right)/\text{aTLV}\right|\times 100\%$$

$$\left|\left(\text{AI}-3\text{DCTVol}-\text{aTLV}\right)/\text{aTLV}\right|\times 100\%$$

The absolute error percentage of pTLV and aTLV is greater than that of AI-3DCTVol and aTLV, with significant differences observed in both the overall and male populations (*p* <0.05). However, no statistical difference was found in the female population (*p* = 0.464), as shown in Table 5.

**TABLE 5:** Absolute error Percentage of aTLV and pTLV, aTLV and AI-3DCTVol.

Characters	N (%)	pTLV and aTLV	AI-3DCTVol and aTLV	*Z*	*p*
Overall	75 (100%)	9.09 (15.58, 3.07)	5.11 (2.92, 11.79)	−2.125	0.034
Male	31 (41.33%)	10.36 (6.34, 16.28)	5.23 (6.34, 16.28)	−2.352	0.019
Female	44 (58.67%)	6.79 (2.04, 13.04)	5.08 (2.70, 12.51)	−0.731	0.464

## Discussion

This study revealed that in all study subjects and subgroups of males and females, the pTLV value was greater than the aTLV value, while the AI-3DCTVol measurement value was less than the aTLV value. The pTLV may be influenced by factors such as age, race, sex, and disease history, resulting in significant numerical deviations (Bellemare et al., 2001; Harik-Khan et al., 2001). The inability to calculate the volume of the upper respiratory tract during the calculation process of AI-3DCTVol possibly led to the small measurement value obtained by AI-3DCTVol. Published sources have indicated that the irregular upper respiratory tract structure often makes it difficult for 3D reconstruction to accurately identify and measure this volume (Sun et al., 2006). The estimated volume of the upper respiratory tract is approximately 300 mL, which is a difference of about 100 mL compared to the difference of 170 mL observed between AI-3DCTVol and aTLV in the results of this study (Blair and Hickam, 1955). It is also possible that this difference is related to segmentation errors when identifying the lung contour boundary using the AI-3D model (Tanaka et al., 2022). Compared to plethysmography examination, it is more difficult to clearly determine the achievement of maximal inspiratory capacity during CT imaging, despite CT technicians instructing subjects to take a full inhalation. Therefore, the team will continue to reduce the occurrence of overfitting phenomena and enhance the modeling accuracy of the AI-3D system. Moreover, compared to the pTLV formula, the intra-group correlation analysis revealed a stronger correlation between the AI-3D measurement and the aTLV benchmark. To further validate these conclusions, an error analysis was conducted and showed that the AI Iterative reconstruction achieved an error control of approximately 5% compared to the approximately 10% error using pTLV, and further demonstrated the improved accuracy in lung volume calculations completed by the AI-3D reconstruction system.

In fact, with the advancement of CT technology, there have been several studies that have applied CT scans to measure lung volumes (Konheim et al., 2016; Yamada et al., 2022). However, the AI-3D method exhibits favorable advantages. AI-based lung segmentation utilizes deep learning technologies to identify lung tissue using a large volume of training data. The AI segmentation logic for lungs extends beyond CT values obtained in the traditional method with more sensitivity, accuracy, and consistency. Traditional CT-based volume calculation is based on algorithms similar to integration, whereby the lung segmentation area of each CT layer is multiplied by the number of layers and layer thickness, potentially leading to significant errors. In contrast, the AI-3D method takes the two-dimensional segmentation and generates a three-dimensional lung model, which provides a higher level of simulation and more vivid lung representation, consequently presenting a more precise lung volume. Such advantages in accurately measuring lung volume possess potential in the matching process of lung volume for lung transplantation donors and recipients.

Lung transplantation is the most effective treatment for end-stage lung disease, aiming to improve patients’ quality of life and extend their survival (Barnard et al., 2013). Matching the donor and recipient size in lung transplantation is essential for the preoperative evaluation of the procedure, as the donor lung must fit within the recipient’s chest cavity. Previous research has shown that improper size matching can lead to surgical complications, primary graft dysfunction (PGD), higher mortality rates, and chronic lung allograft dysfunction (CLAD) (Ganapathi et al., 2017; Li et al., 2021; Montoya et al., 2021). A fast and accurate size matching between the donor’s lung and the recipient’s thoracic cavity can drastically improve the efficiency of the organ transplantation process and prevent the wastage of medical resources.

This study selected healthy individuals as research subjects to verify the accuracy of the AI-3D method to examine the potential application in lung transplantation. The AI-3D technology reconstructed a detailed view of the lungs, which is critical in diagnosing and treating various pulmonary conditions, reducing the need for additional testing or invasive procedures, and ultimately leading to improved treatment planning and patient outcomes. The AI-based reconstruction nature allows patients with severe lung diseases and brain death to accurately calculate their lung volume, which makes the AI-3D method suitable for situations like lung transplantation.

Although this study has possessed certain advantages, it also has several limitations. This study is a single-center study, which lacks data support from multiple clinical institutes. Additionally, the sample size is relatively small and requires an increased sample size for further research validation. Therefore, conducting research using a larger sample size and focusing on the practical application of lung transplantation patients is necessary.

## Conclusion

The AI-3D reconstruction as a convenient method, has significant potential for application in lung transplantation.

## Data Availability

The original contributions presented in the study are included in the article/Supplementary Material, further inquiries can be directed to the corresponding authors.
